# Emerging Strategies for the Biofabrication of Multilayer Composite Amniotic Membranes for Biomedical Applications

**DOI:** 10.3390/ijms241914424

**Published:** 2023-09-22

**Authors:** Mathilde Fenelon, Paul Galvez, Daniel Kalbermatten, Paolo Scolozzi, Srinivas Madduri

**Affiliations:** 1 Division of Oral and Maxillofacial Surgery, Department of Surgery, Geneva University Hospitals, 1205 Geneva, Switzerland; mathilde.fenelon@u-bordeaux.fr (M.F.); paolo.scolozzi@hcuge.ch (P.S.); 2 INSERM, BIOTIS, U1026, Université de Bordeaux, 33076 Bordeaux, France; paul.galvez@u-bordeaux.fr; 3 Plastic, Reconstructive and Aesthetic Surgery Division, Department of Surgery, Geneva University Hospitals and University of Geneva, 1205 Geneva, Switzerland; daniel.kalbermatten@hcuge.ch; 4 Bioengineering and Neuroregeneration Laboratory, Department of Surgery, Geneva University Hospitals and University of Geneva, 1205 Geneva, Switzerland

**Keywords:** amnion, amniotic membrane, electrospinning, biofabrication, tissue engineering, nerve regeneration

## Abstract

The amniotic membrane (AM) is the innermost part of the fetal placenta, which surrounds and protects the fetus. Due to its structural components (stem cells, growth factors, and proteins), AMs display unique biological properties and are a widely available and cost-effective tissue. As a result, AMs have been used for a century as a natural biocompatible dressing for healing corneal and skin wounds. To further increase its properties and expand its applications, advanced hybrid materials based on AMs have recently been developed. One existing approach is to combine the AM with a secondary material to create composite membranes. This review highlights the increasing development of new multilayer composite-based AMs in recent years and focuses on the benefits of additive manufacturing technologies and electrospinning, the most commonly used strategy, in expanding their use for tissue engineering and clinical applications. The use of AMs and multilayer composite-based AMs in the context of nerve regeneration is particularly emphasized and other tissue engineering applications are also discussed. This review highlights that these electrospun multilayered composite membranes were mainly created using decellularized or de-epithelialized AMs, with both synthetic and natural polymers used as secondary materials. Finally, some suggestions are provided to further enhance the biological and mechanical properties of these composite membranes.

## 1. Introduction

The amniotic membrane (AM) is the innermost part of the placenta and forms a sac filled with amniotic fluid in direct contact with the fetus. During pregnancy, the AM plays a crucial role by protecting the fetus from environmental injuries, as well as regulating nutrition and excretion [1]. The AM is a source of stem cells, extracellular matrix proteins, and several growth factors, thereby providing a range of unique biological properties [2]. Furthermore, the AM is a surgical waste obtained after elective cesarean surgery, making it a highly abundant, readily available, and cost-effective biological tissue [3]. The first documented use of AMs in the medical field started in the early 1900s when Davis proposed its application as a skin graft [4]. Over the past century, wound care and ophthalmology have remained the main applications of AMs in medicine by applying AMs as a simple sheet for skin or cornea repair [5,6].

The use of AMs has recently expanded substantially, but with certain limitations to its conventional use, mainly due to its thinness, which make AMs difficult to handle and suture, due to its rapid degradation rate [5,7,8,9,10]. To overcome these limitations and to broaden its potential applications in medicine and tissue engineering, several strategies have been proposed to create hybrid biomaterials derived from AMs [11,12]. First, various modifications of AMs have been proposed to create AM extract, powder, weaving yarns, cross-linked AMs, and solubilized AMs [13,14,15,16,17,18,19]. Importantly, the AM and its derivatives could be combined with secondary natural or synthetic materials and/or additional cells to create novel hybrid scaffolds such as hydrogels, coated AM, and 3D scaffolds, as well as a drug/cell delivery vehicle for the regeneration of various tissues such as muscle, bone, tendon, nerve, gum, cartilage, and heart [7,20,21,22,23,24,25].

Another interesting approach to tuning AM properties is to create multilayer composite membranes by combining the AM sheet with a layer from a secondary material (i.e., mainly polymers) [8,9]. Several strategies have been proposed for this purpose, largely involving the use of electrospinning. Another method consists of attaching a casted layer on top of the AMd [9,12].

This review aims to identify additive manufacturing strategies employed to create multilayer composite constructs derived from AM. In the following sections, we first introduce AM properties, preservation methods, and their application in the field of nerve regeneration. The different strategies employed for engineering AM-based multilayer composite membranes are then reviewed, with a particular emphasis on electrospinning, and their applications for nerve regeneration and other biomedical purposes are discussed.

## 2. Amniotic Membrane Properties

### 2.1. Biological Properties

**Figure 1:**
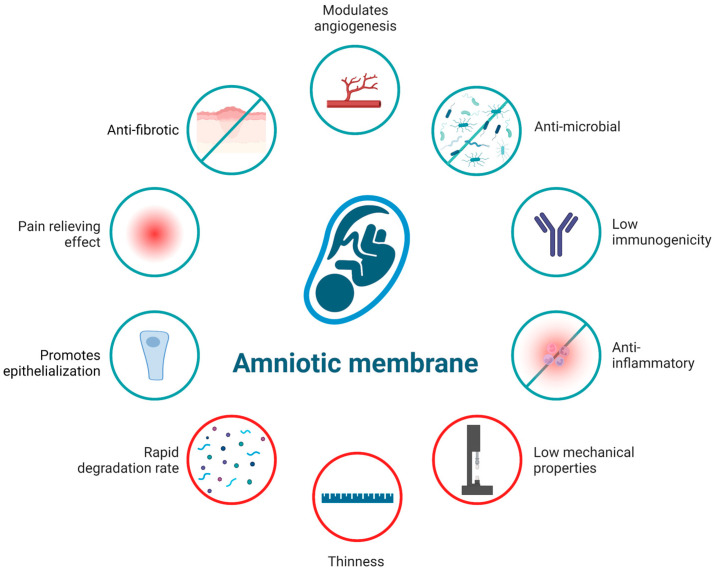
Properties and limitations of the AM highlighting the need to combine AMs with other biomaterials. Created with BioRender.com (accessed on 6 July 2023).

The AM involves a monolayer of epithelium separated from an avascular stroma by a dense basement membrane. AM contains over 226 different growth factors, cytokines, protease inhibitors, and other bioactive molecules [26]. Combined with its low cost and wide availability, AM thus displays several biological properties, which have positioned it as the biomaterial of choice for clinical and tissue engineering purposes (Figure 1) [12]. 

Based on its low immunogenicity and anti-inflammatory effects, the AM is considered to be a suitable tissue for allografts [27]. Its immunomodulatory properties are supported by the low level of expression of HLA class I molecules A, B, and C, which are known to induce a specific immune response by presenting peptide antigens to T cells, and no expression of major histocompatibility class II antigen (HLA-DR). In contrast, AMs contain some immunoregulatory factors, such as HLA-G, which is thought to be involved in the induction of immune tolerance [2,28]. Additionally, there are no lymph vessels and nerves in its structure [9]. The AM also has an anti-inflammatory effect driven by both amniotic epithelial and mesenchymal stem cells, which express anti-inflammatory proteins, such as the interleukin IL-1 receptor antagonist, tissue inhibitors of metalloproteinase (TIMP)-1, -2, -3, -4, and IL-10 and secretes anti-inflammatory factors, such as prostaglandin, transforming growth factor beta (TGF-β), hepatocyte growth factor (HGF), TNF-α, and migration inhibitory factor [29,30]. Some authors also reported that the AM might be considered an anticancer agent with low tumorigenicity, mainly explained by the proapoptotic, antiangiogenic, and immunomodulatory properties of the amnion, but the exact mechanism remains unclear [31,32]. 

The benefit of using the AM for wound healing is also related to its anti-fibrosis, anti-scarring, and anti-adhesive effect [33]. The anti-fibrotic effect of AMs is exerted by the down-regulation of TGF-β, which is responsible for the activation of fibroblasts, stimulates extracellular matrix synthesis, and increases collagen deposition in the wound [34,35,36]. The hyaluronic acid present in the amniotic membrane also inhibits excessive fibrosis [37]. Furthermore, the AM promotes epithelialization from the wound bed and/or wound edge not only by providing physical support for tissue ingrowth but primarily by the production of growth factors that directly and significantly supports epithelization [37]. The AM contains epidermal growth factor (EGF), HGF, and keratinocyte growth factor, necessary for wound healing and regeneration by supporting and promoting the migration, proliferation, and differentiation of epithelial cells [30,34,37,38]. 

Another interesting property of the AM is its antibacterial and antiviral functions by secreting antimicrobial peptides (β-defensins and elafin) that act as a barrier against bacterial infiltration. Its adhesive properties on the wound surface also prevent dead space formation and serous discharge accumulation, which is another mechanism of action against infection, thereby reducing the bacterial load [39]. Its antimicrobial properties are also supported by its inhibitory effect against various bacteria such as streptococcus group A or Staphylococcus aureus [40].

The AM displays a dual effect on angiogenesis by the synthesis of both proangiogenic and anti-angiogenic growth factors. AMs contain proangiogenic growth factors (angiopoietin 2, basic fibroblast growth factor “[bFGF], EGF, HGF, heparin-binding epidermal growth factor, platelet-derived growth factor, placental growth factor, and vascular epidermal growth factor [VEGF]), which trigger the proliferation of endothelial cells and support blood vessels’ neoformation [41]. In contrast, the AM can also have an anti-angiogenic effect by producing several potent anti-angiogenic factors, including endostatin, TIMP-1, -2, -3, and -4, and thrombospondin-1 [29].

Several clinical studies support that the AM relieves pain [2,42,43,44], mainly via the downregulation of pro-inflammatory cytokines such as TNF-α and IL-6 [45], and by acting as a biological dressing that protects the exposed nerve in a wound [3].

Finally, another critical property of decellularized AMs for tissue engineering applications is its ability to support various cell lines’ adhesion, proliferation, and differentiation [12,46]. This is favored by the exposure of the basement membrane of AMs caused by the decellularization process, thereby promoting its ability to support cell adhesion and growth [47].

### 2.2. Physical and Mechanical Properties

During gestation, the fetal membrane must bear the pressure of amniotic fluid and repetitive minor loads caused by contractions [48]. Native AMs are a translucent tissue and their thickness range from 20 μm to 500 μm [39]. The mechanical properties of the AM, such as elasticity, stiffness, and other valuable biomechanical characteristics are additional key elements of its attractiveness for tissue engineering applications [3]. These are attributed to its extracellular matrix components such as proteoglycan, elastin, and collagens [5,10,49]. Its ability to maintain a physiological moist microenvironment also contributes to reducing water loss and promoting wound healing [34]. 

It is noteworthy that mechanical properties may vary within an individual AM depending on the sampling location, i.e., distal or adjacent to the placental disk. It has been established that adjacent human samples of AMs are significantly thicker, stronger, and more stretchable compared to distal samples [50,51]. The mechanical properties of AM are also affected by preservation and sterilization methods [47]. Depending on the targeted application, some authors have reported difficulties related to AM handling and suturing during surgical procedures, as well as the need to tune its mechanical properties by multilayering or associating AMs with other materials [9,52,53]. 

## 3. Amniotic Membrane Collection, Processing, Preservation and Sterilization 

Placenta is usually obtained from healthy pregnant patients undergoing elective cesarean surgery after proper informed consent and rigorous serological screening [54]. After delivery, the collected placenta is placed in a sterile transport medium to avoid drying. The collected placenta is thoroughly washed using a saline sterile solution that may contain antibiotics. The placenta is further processed under aseptic conditions to obtain AM by separating it manually from the underlying chorion [8,34].

While the use of fresh AMs for transplantation in humans has often been described, several processing, preservation, and sterilization methods have been recommended to avoid the potential risk of disease transmission and ensure long-term storage [55]. Most authors now agree on the benefits of using a decellularized membrane [12,46,56,57], thereby avoiding a possible immunogenic response by removing the cells and cellular debris from the AM, while leaving the extracellular structural proteins intact [8]. The decellularization process also exposes the extracellular matrix proteins, making the AM an ideal candidate for tissue engineering by promoting cell adhesion and proliferation [47,58]. The basic methods employed for storing human AMs are cryopreservation, lyophilization, and dry storage [37]. Cryopreservation in glycerol has been the most widely used method for AM preservation [48,59] and is currently used for AM tissue banking. However, it requires a deep-freezing facility, which is an expensive and cumbersome equipment, to freeze and store a high quantity of amniotic tissue at −80 °C. Furthermore, this storage cannot exceed several months. Another challenge lies in the shipment of these cold-storage samples, due to the necessary respect of the cold chain, making transportation at a stable storage temperature difficult [3,48]. Drying methods (i.e., freeze-drying and air-drying) thus marked a turning point in the use of the AM for tissue reconstruction strategies as it proved to be effective and solved the problem of preserving fresh membranes [5]. Lyophilized or freeze-dried AMs can be stored safely for several years at room temperature [3,34,60].

Finally, freeze- and air-drying are usually followed by sterilization of the AM. The most commonly used method is gamma-radiation, allowing storage of the AM for up to 5 years [5,34,61,62,63]. While irradiation seems to have no effect on the biological and physical properties of AMs, it might result in a significant decrease in growth factors [5,47,63]. Other methods of sterilization have been proposed, such as sterilization using a peracetic acid/ethanol mixture or trehalose [48,64].

## 4. Regenerative Properties of Amniotic Membrane to Treat Nerve Injuries

Due to its anti-inflammatory and anti-fibrosis properties, the AM appears to be an ideal biomaterial for nerve repair. Wrapping neurorrhaphy sites with the AM can decrease adhesion and promote functional recovery [65]. Moreover, amnion wraps around nerve injuries may act as a reservoir of neurotrophic factors, such as nerve growth factor, brain-derived neurotrophic factor, neurotrophin, glial-derived neurotrophic factor, and ciliary neurotrophic factor, that can effectively guide nerve regeneration [65,66]. Promising results have been obtained in preclinical and clinical studies using the AM for nerve injury applications as evidenced by the anatomical(axonal regeneration and remyelination) and behavioral (gait analysis and electrophysiological properties) outcome measurements. Most preclinical studies used the same rat sciatic nerve model in which a localized 1–1.5 cm nerve defect or lesion is performed [67,68,69,70,71,72]. They have shown that the use of AMs as a perineural envelope seems suitable for repairing a nerve lesion or defect by reducing fibrosis and perineural adhesions and improving functional recovery in the early phase after a nerve lesion. 

These results are supported by clinical studies [45]. For example, Razdan et al. assessed the efficacy of AMs around nerve bundles during robotic-assisted laparoscopic radical prostatectomy [73]. In this study, 1400 patients were selected who either underwent an intervention with placement of dehydrated AMs allograft wrapped around nerve bundles (n = 700 patients) during robotic-assisted laparoscopic radical prostatectomy or did not (n = 700 patients). Their results highlighted that the use of the AM showed an earlier and overall higher probability of satisfactory potency outcomes 1 year after robotic-assisted laparoscopic radical prostatectomy.

Despite these promising results, it has been suggested that the potential of AMs in nerve regeneration could be further improved. Indeed, due to its fast degradation rate and weak mechanical strength, some authors stated that the AM might not provide sufficient space for nerve regeneration [74]. A recent review of the literature on the use of the AM to treat peripheral nerve defects stated that the best approach would be to keep the AM associated with the chorion layer and use a freeze-dried preservation method in order to maintain all its growth factors and to facilitate membrane handling and storage in the operating room [75]. Similarly, some studies have highlighted the benefit of keeping the AM attached to the chorion in oral surgery [76,77]. Another interesting approach to overcome these limitations would be the use of multilayer composite-based AM.

## 5. Multilayer Composite Amniotic Membranes and Applications

Despite its excellent biological properties, some major challenges have arisen for certain applications of the AM as mentioned above. To overcome some limitations such as its rapid degradation rate and inferior mechanical properties [7], composite multilayer approaches of the AM combined with another material have recently been suggested [9,10]. 

### 5.1. Additive Manufacturing Methods and Electrospinning

In recent years, additive manufacturing, also commonly known as 3D printing, has emerged as a powerful advanced manufacturing technique for producing 3D materials with optimized and multi-functional properties in healthcare [78,79]. Currently, the different additive manufacturing technologies can be mainly classified into material extrusion (such as fused filament fabrication or fused deposition modeling); powder bed fusion; vat photopolymerization (including stereolithography or digital light processing); material jetting; sheet lamination; and melt electrospinning [80,81]. Additive manufacturing is now enabling the development of complex structures of multi-material, functionally graded materials and offers promising perspectives for patient-specific structures for tissue engineering applications [82]. Electrospinning is another processing method to construct 3D tissue engineering scaffolds [78]. This nanofabrication technique is based on the use of high electric voltages to create polymeric fibers with micro-to-nanometer diameters [83].

To the best of our knowledge, 3D printing of materials in direct contact with the AM has never been reported [8]. In contrast, electrospinning is the most commonly used manufacturing strategy to create multilayered composite-derived AMs, thereby reinforcing this natural membrane. Depending on the tissue-specific applications, synthetic and/or natural polymer-based electrospun layers are used to cover the surface of the AM. In this present review, we highlight that, apart from four studies [74,84,85,86], these composite membranes were all performed using decellularized or de-epithelialized AMs. Additionally, we emphasize that all the studies dedicated to these hybrid membranes focused on the AM and no composite membrane using amniochorionic membrane has been performed to date.

### 5.2. Electrospun Multilayered Composite Amniotic Membrane and Secondary Materials

Electrospinning process parameters, such as applied voltage, liquid flow rate, and tip-to-collector distance, are critical for forming electrospun fibers and controlling their diameters [87]. Both synthetic and natural polymers were used as secondary materials to create electrospun multilayered composite AM (Table 1). 

The synthetic electrospun polymers were polycaprolactone (PCL) [74,84,86,88,89,97,98,99], poly(lactic-co-glycolic acid) (PLGA) [90,91,97], poly(lactic acid) (PLA) [97], and poly-(L-lactide-co-E- caprolactone) (PCLC) [85]. They were all biodegradable and biocompatible synthetic polymers. In one study, nanosheets of molybdenum disulfide (MoS2) were integrated into PCL with the purpose of improving the scaffold’s electrical conductivity to target cardiac tissue engineering applications [89].

Silk fibroin was the main natural polymer used to create these electrospun multilayered composite AMs [92,93,94,95,96], mainly to target skin wound healing and regeneration applications. Silk fibroin has excellent biocompatibility and biodegradation properties, with lower mechanical properties [100]. One study reported the use of multilayer composite-based AMs with mixed electrospun PCL/silk fibroin fibers for vascular graft [99]. Silk fibroin was thus combined with PCL to improve the hydrophobicity and degradation rate of PCL, as well as to enhance cell adhesion and proliferation via a more favorable microenvironment than PCL alone and improve early cellular infiltration with degradation. Finally, chitosan was used in one study as it displays biocompatibility, biodegradability, low toxicity, and antibacterial properties [21]. Unfortunately, electrospinning process parameters were not reported in this study and this information was not obtained after contacting the corresponding authors by email.

**Figure 2:**
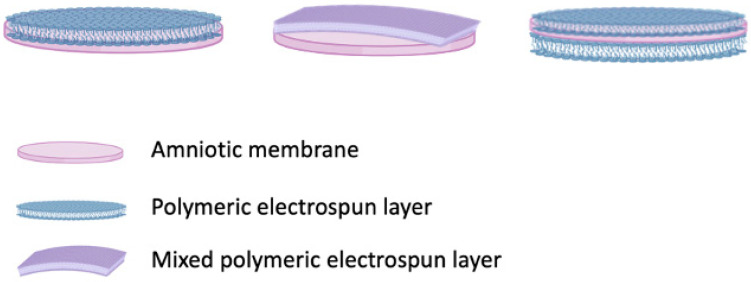
Schematic representation of bilayer composite AMs (combined with a single polymeric electrospun layer or with a mixed polymeric electrospun layer) and three-layer composite AM. Created with BioRender.com (accessed on 17 September 2023).

Composite-based AMs were mainly bi-layer membranes, with the exception of four studies using a three-layer composite membrane where either PCL or PLCL nanofiber layers were applied on both sides of the AM [74,84,85,86]. Interestingly, one clinical study also reported the use of a three-layer composite membrane, which was designed using three different materials: nano-hydroxyapatite, AM, and chitosan. The vast majority of the studies did not specify on which side of the AM the electrospun layer has been applied [88,89,90,92,93,99]. When mentioned, nanofibers were either electrospun on the basement membrane side of the de-epithelized or decellularized AM [95,96] or spread onto the stromal side of the AM [97]. Both sides of the AM were electrospun to construct the three-layer composite membrane [74,84,85,86] (Figure 2).

**Figure 3:**
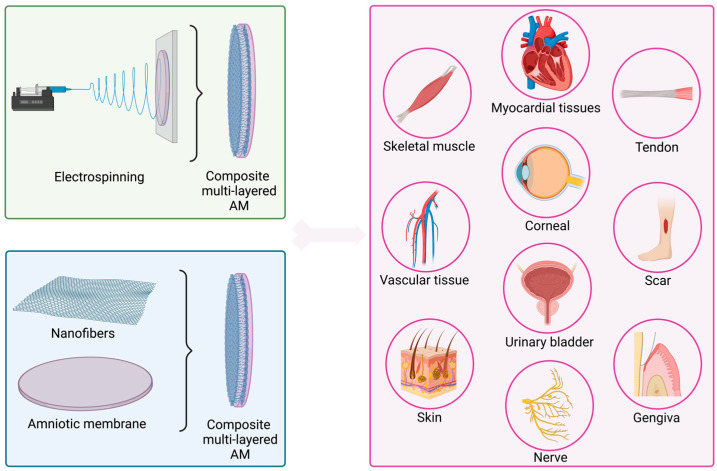
Schematic representation of multilayer composite AMs created by direct electrospinning of a secondary material on AM, or by subsequently assembling AM and a secondary material, and their targeted tissue applications. Created with BioRender.com (accessed on 11 July 2023).

In most studies, the secondary material was directly spun onto the AM, with the AM attached to a mandrel collector or fixed to a drum covered with an aluminum foil. Otherwise, the AM and the nanofiber layer were subsequently assembled by mechanical compression to ensure sufficient contact between both layers [98] (Figure 3).

### 5.3. Characterization of Electrospun Multilayered Composite-Based Amniotic Membrane

#### 5.3.1. Mechanical Characterization

One of the main issues raised by the use of the AM alone in biomedical and tissue engineering fields is its weak mechanical properties. Electrospinning of a polymeric layer onto AM is mostly performed with the purpose of improving the scaffold’s mechanical properties compared to AM alone. Several studies thus assessed the physical and mechanical properties of electrospun multilayered composite-based AMs (Table 2) [84,88,89,90,93,95,97].

The most commonly investigated mechanical features were ultimate tensile strength (UTS) and Young’s modulus, followed by strain to failure and suture retention strength. Studies mainly highlighted the significant increase in composite AM mechanical properties compared to the AM alone [84,88,93,97]. Additionally, the electrospun multilayered composite-based AM showed a significant enhancement of membrane thickness compared to AM alone [88,93].

Only two studies did not report a significant enhancement of AM mechanical properties by adding an electrospun layer. One study showed no significant differences in the UTS and Young’s modulus between the AM and composite AM-PLGA scaffolds. This can be explained by the higher tensile strength of their AM compared to other studies (20.46–7.477 MPa and 26.34–8.991 MPa in dry and wet conditions, respectively) [90]. Arasteh et al. also reported no significant differences in the UTS and Young’s modulus between the AM and composite AM–silk fibroin scaffolds [95].

Interestingly, one study compared three types of multilayered composite-based AMs using three different polymer fibers (PCL, PLA, and PLGA) to target limbal stem cell deficiency applications [97]. Considering the need to balance strength, modulus, and suture retention ability with flexibility and toughness for the application in limbal stem cell transplantation, they concluded that the composite membrane using electrospun PCL was more suitable for this specific application. 

#### 5.3.2. In Vitro and In Vivo Assessment of Electrospun Multilayered Composite-Based Amniotic Membrane

The interest in electrospinning to generate composite-based AM constructs was assessed in vitro in eight studies [88,89,90,93,97] and in vivo in eight preclinical studies [92]. One clinical study reported the use of such electrospun composite-based AM.

Depending on tissue-specific applications, various cell types have been seeded on these designed scaffolds. Rat adipose-derived stem cells (ADSCs) were successfully cultured on aligned and random AM-PCL scaffolds and assessed for muscle tissue engineering applications [88]. The engineered multilayer AM-PCL membranes promoted cell growth and myogenic differentiation of ADSCs. They also enhanced the myotube formation compared to the AM alone, with a significant enhancement of myotube diameters using the aligned fibers pattern [88]. 

Mouse embryonic cardiac cells (mECCs) were seeded on the AM, AM-PCL, and AM-PCL-MoS2 composite membranes to target the cardiac tissue engineering field [89]. An absence of cytotoxicity was observed for the three scaffolds, but the decellularized human AM/PCL-MoS2 scaffold revealed more expansion, elongation, and attachment of mECCs. In addition, the results showed that the reinforcement of the AM using electrospun PCL-MoS2 fibers increased the expression, maturation, and upregulation of main cardiomyocyte genes in mECCs. 

Hasmad et al. seeded human skeletal muscle cells on the AM and AM-PLGA scaffolds and observed a similar viability and migration rate [90]. They also reported that skeletal muscle cells seeded on AM-PLGA scaffolds were oriented along the alignment of the electrospun PLGA fibers, whereas cells seeded on AM displayed random orientation, thereby guiding the orientation and migration of skeletal muscle cells owing to their aligned topography. In a further study, they investigated the proangiogenic potential of the same skeletal muscle cell-seeded composite scaffolds according to three different thicknesses of PLGA fibers [91]. Thus, they compared in vitro the angiogenic paracrine effects of (i) these three skeletal muscle cell-seeded composite scaffolds, (ii) skeletal muscle cell-seeded AM, and (iii) non-seeded skeletal muscle cells on human umbilical vein endothelial cells (HUVECs). The most promising results were achieved with the skeletal muscle cell-seeded composite scaffold with the thickest fibers, with significantly elevated levels of proangiogenic factors, which induced a higher migration capacity in HU-VECs and formed a longer and more elaborated capillary-like network compared to non-seeded skeletal muscle cells.

Composite-based AM was also investigated for ophthalmological applications. Liu et al. reported the potential of AM-PCL composite membrane in the treatment of limbal stem cell (LSC) deficiency by demonstrating its ability to support rabbit LSC attachment and growth [97]. Zhou et al. then compared in vivo the seeded and non-seeded AM-PCL composite membranes versus seeded and non-seeded AM in a rabbit LSC deficiency corneal epithelial defect model [98]. They observed that the AM-PCL composite membrane maintained the pro-regenerative and immunomodulatory properties of AM, promoted LSC survival, retention, and organization, and improved re-epithelialization of the defect area while reducing inflammation and neovascularization.

To assess the potential of AM–silk fibroin membrane to accelerate post-injury neovascularization, Gholipourmalekabadi et al. seeded adipose tissue-derived mesenchymal stem cells (AT-MSCs) on AM and AM–silk fibroin and investigated the secretion of pro-angiogenic factors [93]. Both membranes were cytocompatible. Moreover, they observed an increased expression of two main pro-angiogenesis factors (VEGF and bFGF) when AT-MSCs were cultured on the electrospun multilayered composite-based AM for 7 days compared to the AM alone, thereby suggesting its interest in a clinical setting for skin regeneration. In a second study, the authors further investigated the potential of this membrane to support wound healing in vivo [92]. For this purpose, AM and AM–silk fibroin alone or seeded with AT-MSCs were implanted in full-thickness burn wounds in mice. Healing efficacy and scar formation were evaluated at 7, 14, and 28 days post-implantation. They observed that the wound healing process was accelerated by silk fibroin and a significant reduction in the post-burn scar using seeded AM–silk fibroin. In a third study, they assessed the potential of this same composite AM–silk fibroin to modulate post-wound hypertrophic scar formation compared to the AM alone and a control group (i.e., no graft) in a rabbit ear model [94]. Results highlighted the potential of this composite membrane to act as an efficient anti-scarring wound dressing material via a significant decrease in collagen deposition and expression, as well as the increased expression and deposition of MMP1 in the wound bed compared to the AM and control groups. Another team also investigated the potential of composite AM–silk fibroin membranes for skin tissue engineering strategies. First, Arasteh et al. showed that the AM–silk fibroin membrane was a suitable scaffold to support mouse embryonic fibroblasts’ attachment and proliferation [95]. To further assess this composite membrane for stem cell-based skin wound healing and regeneration, they investigated the potential of menstrual blood-derived stem cells (MenSCs) to generate keratinocytes when seeded on this membrane [96]. For this purpose, the seeded MenSCs over AM–silk fibroin scaffold were co-cultured with foreskin keratinocytes to induce MenSCs differentiation into keratinocytes. Results showed that newly differentiated keratinocytes expressed the keratinocytes’ specific markers at a higher level when cells were seeded over an AM–silk fibroin scaffold compared to a conventional 2D culture system. Based on these findings, the authors stated that a bi-layer AM–silk fibroin scaffold is an efficient natural construct to generate keratinocytes from MenSCs for stem cell-based skin wound healing and regeneration.

The use of the composite-based AM was also investigated for vascular applications. The AM with mixed electrospun PCL/silk fibroin fibers (AM-PCL/silk fibroin) was applied in a rat aortic grafting model and compared to a decellularized porcine small intestinal submucosa-integrated PCL/silk fibroin graft and an autologous aorta [99]. The AM-PCL/silk fibroin graft induced rapid functional endothelialization resisted collagen deposition and showed a lower inflammatory response and foreign body reaction 4 weeks after implantation. They also observed that the AM-PCL/silk fibroin graft maintained patency by progressively stabilizing the remodeling structure towards that of the native aorta.

A composite multilayered AM was also investigated in the field of reconstructive urology as a graft for urinary bladder augmentation in a rat model [85]. For this purpose, rats underwent hemicystectomy and their bladders were augmented using the AM-PLCL composite membrane. They reported effective regeneration of the urothelial and smooth muscle layers using the composite membrane. Mechanical properties of the reconstructed bladder wall were then compared to the intact bladder wall. Results showed that Young’s modulus of the regenerated bladder wall was significantly higher than the native bladder wall, certainly related to the presence of fibrotic lesions and locally disturbed cytoarchitecture within the augmented wall. Unfortunately, it must be emphasized that there was no control group in this study.

A three-layer composite scaffold made from PCL and AM was assessed in vitro and in vivo for the prevention of post-surgical tendon adhesion [84]. First, the composite membrane was seeded with tenocytes and fibroblasts. Results showed that both the AM and AM-PCL were biocompatible and promoted the adhesion and proliferation of tenocytes and fibroblasts by up-regulating the phosphorylation of ERK1/2 and SMAD2/3. The composite membrane was then assessed in vivo in a rabbit tendon repair model. The wound was sutured directly (control) or either the freeze-dried AM or the composite AM-PCL was wrapped around the suture. Results showed that the composite AM-PCL membrane effectively isolated the exogenous adhesion tissue and promoted endogenous tendon healing. 

Finally, to our knowledge, there is only one clinical study reporting the use of an electrospun composite multilayer-based AM in patients [21]. In this study, a three-layer composite based-AM (chitosan-AM-hydroxyapatite) was applied to treat gingival recession defects and compared to the AM in nine patients. Interestingly, the three-layer membrane was designed so that the hydroxyapatite layer could be applied in contact with the bone, while the chitosan layer was applied to interface with the gum. Clinical parameters were significantly improved in both groups, while linear bone growth assessed by radiographic assessment was significantly enhanced only in the three-layer composite AM group. This could be explained by the osteoconductive properties of hydroxyapatite. 

#### 5.3.3. Application of Electrospun Multilayered Composite-Based Amniotic Membrane for Nerve Regeneration

The anti-adhesive effect of the composite multilayer AM was also investigated in vivo for nerve repair strategies in two pre-clinical studies. First, a three-layer composite AM-PCL membrane was applied in a rat model of sciatic nerve compression to determine whether this composite membrane can exert beneficial effects in a chronic nerve injury animal model [86]. In this study, AM-PCL was compared to (i) a medical chitosan hydrogel group and (ii) the absence of treatment. The AM-PCL membrane significantly reduced peripheral nerve adhesion after 8 weeks compared to the two other groups and promoted the rapid recovery of nerve conduction. Furthermore, the immunohistochemical analysis revealed fewer pro-inflammatory M1 macrophages and more Schwann cells in the AM-PCL group. They also observed an increased expression level of nerve growth factor, while the expression levels of types-I and -III collagen were reduced in rats treated with AM-PCL. In a second study, the effect of the AM-PCL membrane was further assessed in an acute nerve injury animal model [74]. A rat sciatic nerve transection model was used in order to determine its impact on nerve regeneration and scarring formation at the nerve repair level for nerve function recovery. After transversally cutting the sciatic nerve, epineural sutures were performed to reconnect the proximal and distal nerve stumps (control group), or the AM-PCL membrane was wrapped around the epineural sutures of the repaired site. Results showed that AM-PCL is a promising candidate to improve nerve regeneration and prevent fibrosis after nerve repair by significantly decreasing the degree of nerve adhesion, collagen deposition, and intraneural macrophage invasion after 4 weeks. Moreover, AM-PCL increased the expression of anti-inflammatory cytokines, while the expression of pro-inflammatory cytokines decreased. After 8, 12, and 16 weeks, the AM-PCL membrane significantly improved functional recovery compared to the control group as demonstrated by the sciatic functional index. The authors also observed that the AM-PCL membrane promoted nerve Schwann cell proliferation and axon regeneration, and reduced the time of muscle denervation after 16 weeks. 

## 6. Future Perspectives

This review highlights the growing number of studies investigating multilayer composite AMs using both synthetic and natural polymers and their promising results. However, in our opinion, further studies are needed to provide clear guidance for researchers in selecting appropriate secondary materials according to the targeted application. 

We also highlight that many approaches are still possible to fine-tune the properties of these membranes and broaden their applications. (1) One possibility would be to perform a multilayer composite membrane using the amnion/chorion membrane. Keeping the amnion attached to the chorion will enhance the biological properties of the composite membrane. Notably, the chorion contains several growth factors in higher amounts [101] and has superior antibacterial properties compared to the AM, thereby improving its antimicrobial effect [102]. (2) Another possibility would be to combine the polymer with other compounds [103]. Since the polymers are processed in liquid formulations during electrospinning, organic and inorganic compounds such as hydroxyapatite could be easily included [104]. The addition of hydroxyapatite might be useful in various fields, such as bone tissue engineering. (3) A third interesting strategy to consider would be to manufacture composite membranes using 3D-printing technologies, whether or not combined with electrospinning. Three-dimensional printing, such as fused deposition modeling, could further enhance the composite membrane’s mechanical properties [103,105]. These membranes could thus be applied in various fields such as load-bearing applications. (4) The AM with aligned fibers loaded with growth factors, neurotrophic factors, and cytokines would enhance their applications in the field of aligned tissue grafts such as peripheral nerves, tendon and muscle tissues, and hollow organs. 

## 7. Conclusions

While wound care and ophthalmology have remained the main applications of the AM in medicine over the past century, the use of AMs has recently expanded substantially. To improve AM properties and broaden its applications, multilayer composite approaches have gained an increasing interest for biomedical and tissue engineering purposes. While 3D-printing technologies have never been applied to AM, electrospinning is the most commonly used strategy to create multilayered composite-derived AM. For this purpose, the AM was mainly used decellularized or de-epithelialized. These composite membranes mostly showed biocompatibility and significantly enhanced mechanical properties. They were also successfully implanted in vivo and showed promising results for various targeted tissue applications, such as nerve regeneration. 

## Figures and Tables

**Table 1:** Electrospinning parameters and secondary materials used to design multilayer composite AMs and their targeted applications.

References	Electrospun Secondary Material	Applied Voltage	Polymer Flow Rate	Deposition Distance	Post-Treatment/Sterilization Process	Targeted Applications
Hadipour et al., 2021 [88]	PCL	22 kV	1 mL/h	16 cm	Disinfection via 70% *v/v* alcohol/water solution (70:30) for 1 h and rinsed with culture medium	Muscle tissue engineering
Nazari et al., 2022 [89]	PCL ± MoS2	20 kV	0.3 mL/h	20 cm	Sterilized using 70% filtered ethanol for 2 h and UV radiation for 20 min	Cardiac tissue engineering
Hasmad et al., 2018 [90]	PLGA	15 kV	0.3 mL/h	15 cm	Air-dried + UV-irradiated for 30 min	Aligned tissue regeneration
Hasmad et al., 2022 [91]		7.5 kV			Air-dried + UV-irradiated for 40 min	Ischemic tissues
Gholipourmalekabadi et al., 2018a [92]	Silk fibroin	18 kV	0.3 mL/h	15 cm	Treatment 70% ethanol for 1 h and subsequently dried under vacuum before storage at 4 °C	Skin substitute
Gholipourmalekabadi et al., 2018b [93]						
Gholipourmalekabadi et al., 2019 [94]						
Arasteh et al., 2016 [95]	Silk fibroin	15–20 kV	0.2 mL/min	8–18 cm	Incubation in methanol for 1 h, distilled water rinse, freeze-dried, and stored at −20 °C	Skin wound healing
Fard et al., 2017 [96]		18 kV		8 cm	Incubation in methanol for 1 h, distilled water rinse and air-dried	
Liu et al., 2018 [97]	PLGA	6.8 kV	0.5 mL/h	NR	Freeze-dried and then stored at −20 °C	Limbal stem cell deficiency
	PLA	16 kV				
	PCL	12 kV	2.5 mL/h			
Zhou et al., 2019 [98]						
Liu et al., 2022 [99]	PCL + silk fibroin	18–22 kV	1.2 mL/h	15 cm	NR	Vascular graft
Liu et al., 2020 [84]	PCL	13 kV	1.0 mL/h	15 cm	Air-dried	Tendon adhesion
Dong et al., 2020 [86]					Air-dried + UV radiations	Nerve injuries
Bai et al., 2022 [74]					Air-dried + sterilized by cobalt 60 irradiation	Nerve injuries
Adamowicz et al., 2016 [85]	PLCL	15 kV	0.500 μL/h	20 cm	NR	Reconstructive urology
Dhawan et al. [21]	Chitosan HA	NR	NR	NR	NR	Gingival recession

Electrospinning parameters and secondary materials used to design multilayer composite AMs and their targeted applications. DMF: dimethylformamide; HA: hydroxyapatite; MoS2: Molybdenum disulfide; NR: not reported; PCL: polycaprolactone; PLA: poly(lactic acid); PLCL: poly-(L-lactide-co-E-caprolactone); PLGA: poly(lactic-co-glycolic acid); RPM: rotation per minute.

**Table 2:** Composition and mechanical characterization of electrospun multi-layer composite-based AM compared to AM.

References	Layers Number and Composition	Mechanical Testing	Main Results (Mean ± SD)
Hadipour et al., 2021 [88]	Bi-layer: 1-AM 2-PCL	50 mm × 10 mm shape strips: -UTS (MPa) -Young’s modulus (MPa)	-Significant enhancement of UTS for both aligned (0.41 ± 0.107) and random (0.26 ± 0.116) AM-PCL compared to AM (0.16 ± 0.035) (MPa) -Similar tensile strength of both aligned and random AM-PCL and AM -Significant enhancement of Young’s modulus for both aligned (2.48 ± 0.216) and random (1.34 ± 0.207) AM-PCL compared to AM (0.976 ± 0.028) (MPa)
Nazari et al., 2022 [89]	Bi-layer: 1-AM 2-PCL Or 1-AM 2-PCL-MoS2	30 mm × 10 mm shape strips: -UTS (MPa) -Young’s modulus (MPa) -Strain at the breakpoint (%)	-UTS: 1.87 ± 0.16 MPa (AM), 3.14 ± 0.11 (AM-PCL) and 4.33 ± 0.23 MPa (AM-PCL-MoS2) -Young’s modulus: 45.95 (AM), 47.21 (AM-PCL) and 49.87 MPa (AM-PCL-MoS2) -Fracture point: 4.87% (AM), 6.46% (AM-PCL) and 9.39% (AM-PCL-MoS2)
Hasmad et al., 2018 [90]	Bi-layer: 1-AM 2-PLGA	3 mm × 10 mm shape strips: -UTS (MPa) -Young’s modulus (MPa)	-No significant difference in the UTS between AM and AM-PLGA scaffolds (in dry or wet conditions) -No significant difference in Young’s modulus between AM and AM-PLGA scaffolds (in dry or wet conditions) -Significant reduction in Young’s modulus with wet AM and AM-PLGA scaffolds compared with dry AM and AM-PLGA scaffolds
Gholipourmalekabadi et al., 2018a [92]	Bi-layer: 1-AM 2-Silk fibroin	20 mm × 10 mm shape strips: -Maximum load value (N) -Suture retention strength (mN) -Strain deflection at break (mm)	-Significant enhancement of maximum load value for AM–silk fibroin (1.9 ± 0.18 N) compared to AM (1.3 ± 0.17 N) -Significant enhancement of suture retention strength for AM–silk fibroin (692 ± 31 mN) compared to AM (512 ± 63 mN) -Significant enhancement of strain deflection at break for AM–silk fibroin (8.5 ± 0.33 mm) compared to AM (7.3 ± 0.49 mm)
Arasteh et al., 2016 [95]	Bi-layer: 1-AM 2-Silk fibroin	35 mm × 12–15 mm shape strips: -UTS (MPa) -Young’s modulus (MPa)	-De-epithelialization significantly increased the UTS and Young’s modulus of AM from 16.14 MPa and 68.46 MPa to 25.69 MPa and 108.03 MPa, respectively. -No significant difference in the UTS between AM and AM–silk fibroin -No significant difference in Young’s modulus between AM and AM–silk fibroin
Liu et al., 2018 [97]	Bi-layer: 1-AM 2-PCL Or 1-AM 2-PLA Or 1-AM 2-PLGA	10 mm × 10 mm shape strips: -UTS (MPa) -Elastic modulus (MPa) -Strain to failure (%) -Toughness 20 mm × 10 mm shape strips: -Suture retention strength (mN)	-Significant enhancement of UTS for the three composite membranes compared to AM alone -Significant enhancement of elastic modulus for the three composite membranes compared to AM alone -Significant enhancement of strain to failure with PCL-AM membrane compared to AM -Significant enhancement of toughness for the three composite membranes compared to AM alone -Significant enhancement of suture retention strength for the three composite membranes compared to AM alone
Liu et al., 2020 [84]	Three-layer: 1-PCL 2-AM 3-PCL	50.0 × 5.0 mm^2^ shape strips: -UTS (MPa) -Elastic modulus (MPa) -Strain to failure (%)	-Significant enhancement of UTS, elastic modulus, and strain to failure with the composite membrane compared to AM alone

AM: amniotic membrane; MoS2: molybdenum disulfide; PCL: polycaprolactone; PLA: polylactic acid; PLGA: poly(lactic-co-glycolic acid); SD: standard deviation; UTS: ultimate tensile strength.

## Data Availability

All datasets related to this article will be provided upon request.
